# Supplementary material for: Isolation and Characterization of the GmMT-II Gene and Its Role in Response to High Temperature and Humidity Stress in Glycine max
Source: Plants (Basel). 2022 Jun 3;11(11):1503. doi: 10.3390/plants11111503 (PMC9182806; doi:10.3390/plants11111503)
Supplement: Supplementary file 1 [file plants-11-01503-s001.zip › plants-1719848-supplementary.pdf]

Table S1. The primers used in allexperiments.

Primer names	Primer sequence (5′-3′)	Experiments
ORF-GmMT-II-F	AGAGAGAAAAATGGCTGATACAAGT	segregation of genes
ORF-GmMT-II-R	AAATCACATCCACAATTTGAACA	
pBT3Gm1-MMP-F	TTGGCCATTACGGCCATGACTCTCCGCAACCACC	Yeast Two-Hybrid Rotation Validation
pBT3Gm1-MMP-R	AAGGCCGAGGCGGCCCCGGGGTTGATACCATAGAGCTTTCG	
pPR3N-GmMT-F	TTGGCCATTACGGCCATGGCTGATACAAGTGGAG	
pPR3N-GmMT-R	AAGGCCGAGGCGGCCCAGTGCGGCAAGAGGCACATGAGCA	
nE-Gm1-MMP-F	GCTTCGAATTCTGCAGTCGACATGACTCTCCGCAACCACC	BiFC experiments
nE-Gm1-MMP-R	GACTCTAGATCAGGTGGATCCGGGGTTGATACCATAGAGC	
cE-GmMT-F	GCTTCGAATTCTGCAGTCGACATGGCTGATACAAGTGGAG	
cE-GmMT-R	GACTCTAGATCAGGTGGATCCAGTGCGGCAAGAGGCACAT	
nEYFP-F	GAAGAACGGCATCAAGGT	
nEYFP-R	CGACAGGTTTCCCGACTG	
cEYFP-F	CACTACCAGCAGAACACCC	
cEYFP-R	CAGCTATGACCATGATTAC	
Gm1-MMP-S-F	TTGGCCATTACGGCCATGACTCTCCGCAACCACC	Interaction site research
Gm1-MMP-S-R	AAGGCCGAGGCGGCCCCTAGAATTGCAAGAGCAACCAAGAG	
Gm1-MMP-P-F	TTGGCCATTACGGCCTATTTTCTTGCCACCTCAC	
Gm1-MMP-P-R	AAGGCCGAGGCGGCCCCGGTATAGTCCGAGATCATGCCAAA	
Gm1-MMP-C-F	TTGGCCATTACGGCCTTCTTCAAAGACATGCCGC	
Gm1-MMP-C-R	AAGGCCGAGGCGGCCCCGGGGTTGATACCATAGAGCTTTCG	
GmMT-N-F	TTGGCCATTACGGCCATGGCTGATACAAGTGGAG	
GmMT-N-R	AAGGCCGAGGCGGCCCGCACCTGCAAGTGGAACCACCAGT	

GmMT-C-F	TTGGCCATTACGGCCACAAGTGTCGGCATG ACAA	
GmMT-C-R	AAGGCCGAGGCGGCCCAGTGCGGCAAGAG GCACATGAGCA	
qRT-GmMT-II-F	GCAGTGAGACCGGTGGTAATA	
qRT-GmMT-II-R	AGTTCCAGAAGCCGCAGTC	RT-qPCR
Actin-F	CCTCAACCCAAAGGTCAACAG	
Actin-R	GACCAGCGAGATCCAAACGAA	
pA7-GmMT-II-F	CACCATCACCATCACGCCATGATGGCTGAT ACAAGTGGAG	
pA7-GmMT-II-R	CACTAGTACGTCGACCATGGCAGTGCGGCA AGAGGCACAT	Subcellular localization
pA7-F	AAGCAATCAAGCATTCTACTTCTAT	
pA7-R	GGTAGCGGCTGAAGCACTG	
